# Increasing objectively measured sedentary time increases clustered cardiometabolic risk: a 6 year analysis of the ProActive study

**DOI:** 10.1007/s00125-013-3102-y

**Published:** 2013-11-06

**Authors:** Katrien Wijndaele, Gillian Orrow, Ulf Ekelund, Stephen J. Sharp, Søren Brage, Simon J. Griffin, Rebecca K. Simmons

**Affiliations:** 1 MRC Epidemiology Unit, Institute of Metabolic Science, University of Cambridge School of Clinical Medicine, Box 285, Cambridge Biomedical Campus, Cambridge, CB2 0QQ UK; 2 Department of Public Health and Primary Care, University of Cambridge, Cambridge, UK; 3 Department of Sport Medicine, Norwegian School of Sport Sciences, Oslo, Norway

**Keywords:** Adiposity, Cardiovascular disease risk, Longitudinal study, Moderate-to-vigorous physical activity, Sedentary behaviour, Television viewing

## Abstract

**Aims/hypothesis:**

We aimed to quantify the associations between change in objectively measured sedentary and moderate-to-vigorous physical activity (MVPA) times and self-reported television viewing over 6 years and change in a clustered cardiometabolic risk score (CCMR), including and excluding waist circumference (CCMR without adiposity component, CCMR_*no adip*_), and its individual components, among the adult children of people with type 2 diabetes.

**Methods:**

In 171 adults (mean ± SD age 42.52 ± 6.30 years; 46% men) with a parental history of diabetes (ProActive UK), physical activity accelerometer measures and self-reported television viewing were assessed at baseline and a mean ± SD of 6.27 ± 0.46 years later. Associations between change in sedentary time, MVPA time and television viewing and cardiometabolic risk and mediation by adiposity change were examined by multiple linear regression and the product of coefficients method, respectively.

**Results:**

Greater increases in sedentary time (h/day) were associated with larger increases in clustered cardiometabolic risk (CCMR: 0.08 [95% CI 0.01, 0.15]; CCMR_*no adip*_: 0.08 [0.01, 0.16]) and triacylglycerol (0.15 [0.01, 0.29]), independent of baseline sedentary and MVPA times, change in MVPA time and other confounders. No evidence was found for mediation by change in waist circumference and BMI for the associations with CCMR_*no adip*_ and triacylglycerol. Greater increases in MVPA time (h/day) were associated with larger decreases in waist circumference (−3.86 [−7.58, −0.14]), independently of baseline MVPA and sedentary times, change in sedentary time and other confounders. Television viewing was not independently associated with any of the cardiometabolic outcomes.

**Conclusions/interpretation:**

Increasing sedentary time is independently related to increasing clustered cardiometabolic risk and triacylglycerol in adults at high risk of developing diabetes. Strategies to prevent diabetes might target reducing sedentary time.

*Trial registration* ISRCTN61323766

**Electronic supplementary material:**

The online version of this article (doi:10.1007/s00125-013-3102-y) contains peer-reviewed but unedited supplementary material, which is available to authorised users.

## Introduction

Central obesity, dysglycaemia, dyslipidaemia and hypertension are closely related cardiometabolic abnormalities that cluster in a large proportion of the adult population worldwide. This cluster of factors substantially increases the risk of developing type 2 diabetes, as well as cardiovascular and other chronic diseases [1, 2]. Insufficient moderate-to-vigorous physical activity (MVPA) is a recognised contributor to elevated cardiometabolic risk levels [3] and has been targeted by lifestyle interventions in adults at high risk of type 2 diabetes. However, such interventions have had limited success, resulting in relatively modest changes in MVPA [4].

Excessive time spent sedentary is associated with an increased cardiometabolic risk, independent of time spent in MVPA, and has been suggested as a potential target for lifestyle interventions [5–9]. Sedentary behaviour represents any waking behaviour characterised by an energy expenditure of ≤1.5 metabolic equivalents (METs) while in a sitting or reclining posture [10]. Adults, on average, spend more than 7 h of the waking day sedentary [11], an amount that is relatively independent of the time spent in MVPA [12]. However, there is currently insufficient evidence to establish a longitudinal association between sedentary time and cardiometabolic risk [13]. The few longitudinal studies examining this hypothesis [14–17] have mainly focused on self-reported television viewing as a marker of leisure-time sedentary behaviour [15, 16]. Although television viewing is a potentially interesting intervention target, it is only one aspect of sedentary time and its association with cardiometabolic risk may differ from that of overall sedentary time [7, 18].

Because individuals at high risk of diabetes experience difficulties in increasing their MVPA [4], identifying additional behavioural targets should inform the development of future preventive interventions in these populations. Longitudinal investigations incorporating objectively quantified time spent sedentary and in MVPA, as well as television viewing time, can provide novel insights into whether, and to what extent, changing these behaviours will independently impact cardiometabolic risk. Furthermore, elucidating whether these associations are predominantly explained by a change in adiposity or by alternative physiological processes will provide insight into the mechanism underlying these associations [19]. Central obesity is regarded as an important aetiological component in the clustering of elevated cardiovascular risk factors and may therefore act as a mediator [20].

We aimed to estimate the independent associations between changes in objectively measured time spent sedentary, in MVPA and in self-reported television viewing over 6 years and changes in clustered and individual cardiometabolic risk factors in adults with a parental history of type 2 diabetes. We also examined whether these associations were mediated by a change in central or general adiposity.

## Methods

We performed a cohort analysis of the ProActive trial, the design of which is described in detail elsewhere [21]. In short, ProActive evaluated the efficacy of a theoretical, evidence- and family-based intervention programme that aimed to increase physical activity levels in adults who were at high risk of type 2 diabetes by virtue of having a parent with the disease. Out of 465 eligible individuals aged 30–50 years, 365 were randomly assigned to one of three interventions delivered over 1 year. There was no significant difference in accelerometry-determined physical activity between the three trial arms at 1 year [22]. Consequently, a cohort analysis was conducted after pooling data from the three trial arms. Participants with valid accelerometry data at 1 and 7 years of follow-up (referred to below as baseline and follow-up, respectively) (*n* = 202) constituted the sampling frame for this study, as this provided the optimal combination in terms of follow-up time and number of participants with complete accelerometry data. A final sample of 171 participants was included in analyses, after excluding those with missing cardiometabolic variables (*n* = 23), covariates (*n* = 6) or unrealistic data (television viewing time > objectively measured sedentary time; *n* = 1). We also excluded 1 person who became a wheelchair user during follow-up. All participants provided written informed consent and ethical approval was obtained from the Cambridge Central Research Ethics Committee (reference number: 09/110308/3). All investigations were carried out in accordance with the principles of the Declaration of Helsinki.

### Outcome: cardiometabolic risk

Data-collection procedures were the same at baseline and follow-up [21, 23]. In brief, participants attended the study centre, following an overnight fast, where a venous blood sample was collected. Standardised procedures were used to determine fasting plasma glucose (hexokinase method), triacylglycerol and HDL-cholesterol (standard enzymatic methods) and serum insulin levels (1235 AutoDELFIA immunoassay system, DAKO, Ely, UK) [23]. Trained personnel measured weight on standard calibrated scales and height using a rigid stadiometer with participants wearing light clothing. Waist circumference was measured twice at the mid-point between the lower costal margin and the level of the anterior superior iliac crests. Systolic and diastolic blood pressures were measured in seated participants three times at 1 min intervals using an automated Accutorr sphygmomanometer (Accutorr, Cambridge, UK). The average of multiple assessments was used.

A clustered cardiometabolic risk score (CCMR) was computed [24, 25], incorporating indicators of central obesity (waist circumference), dyslipidaemia (triacylglycerol and HDL-cholesterol), hypertension (systolic and diastolic blood pressure) and hyperglycaemia (fasting plasma glucose and serum insulin). Individual variables were standardised (i.e. *z* scores were computed: *z* = [value − mean]/SD), after normalising (log_10_) triacylglycerol, glucose and insulin. Sex-specific baseline means and SDs from all participants with complete data for each cardiometabolic variable at baseline were used to standardise the baseline and follow-up variables. Subsequently, individual *z* scores were summed, after inverting HDL-cholesterol *z* scores and averaging systolic and diastolic blood pressure *z* scores, and divided by the number of variables included. The CCMR was also calculated without the adiposity component (CCMR_*no*_ _*adip*_), which allowed us to examine a potential mediation effect by change in adiposity for the associations with clustered cardiometabolic risk.

### Exposure: objectively measured time spent sedentary and in MVPA, and self-reported television viewing time

Physical activity was measured using the ActiGraph accelerometer (Manufacturing Technology, Fort Walton Beach, FL, USA), worn for 4 consecutive days at baseline and follow-up, as previously described [23]. ActiGraph models 7164 and GT1M were used at baseline and follow-up, respectively, providing a relatively comparable intensity classification of free-living activities [26, 27]. Continuous strings of zeros lasting for longer than 60 min were classified as non-wear time. Participants with fewer than three valid wear days (each including >500 min of valid wear time) at baseline or follow-up were excluded (*n* = 21). Sedentary time was defined using a cut-off of <100 counts/min [28], and time spent in MVPA using a cut-off of ≥1,952 counts/min [29]. A customised program (MAHUffe; www.mrc-epid.cam.ac.uk) was used for data cleaning and reduction. Time spent watching television and video (h/day) was self-reported using the EPAQ2 questionnaire, which has high repeatability and sufficient validity for ranking individuals [30].

### Confounding variables

Participants completed another standardised questionnaire, which asked about smoking (current/former/never), age at finishing full-time education as a marker of socioeconomic status (SES; younger/older than 16 years) and medication use for dyslipidaemia, hypertension or dysglycaemia (yes/no).

### Statistical analysis

All analyses were conducted using IBM SPSS Statistics 19 (SPSS, Chicago, IL, USA) and Stata 12.0 (Stata, College Station, TX, USA), and statistical significance was set at *p* < 0.05. Descriptive characteristics (means ± SD) were compared between baseline and follow-up (paired *t* tests), and between sexes and those who were included and excluded in the current study (independent *t* tests). Changes in exposure and outcome variables were calculated as follow-up minus baseline. Change in fasting plasma glucose was normalised (log_10_).

Multiple linear regression was used to examine the association between change in the exposure variables (objectively measured time spent sedentary and in MVPA, and television viewing time) with change in cardiometabolic risk over 6 years. The results are presented as unstandardised and standardised regression coefficients (95% CI), following standardisation of both exposure and outcome variables. Unstandardised regression coefficients enable inference of the effect size in the original units of the exposure and outcome variables, whereas standardised regression coefficients enable direct comparison of the effect sizes across the exposure and outcome variables, as they are all expressed in SD units. Models were initially (Model A) adjusted for age, sex, SES, baseline exposure and the cardiometabolic risk variables under study; baseline and change in smoking status; change in monitor wear time and in dyslipidaemia, hypertension or dysglycaemia medication (as applicable); and follow-up time. Subsequently, we added baseline and change in objective MVPA (when change in sedentary time or television viewing was the exposure) or in sedentary time (when change in MVPA was the exposure) to these models (Model B). The variance inflation factor did not exceed 5 in any of the models, indicating absence of multicollinearity. No significant interactions between any of the exposures and sex were found, so the results are shown for both sexes combined. Including intervention arm in the analyses resulted in very similar associations in terms of their magnitude and direction. This covariate was therefore not included in the final models.

The mediating role of change in central and general adiposity was examined by means of the product of coefficients (a*b) method by MacKinnon et al [31], based on the regression coefficient between the exposure and mediator (*a*) and between the mediator and outcome (*b*). ‘*a*’-path coefficients were adjusted for all covariates in Model B. ‘*b*’-path coefficients were adjusted for all covariates in Model B, including baseline and change in the relevant exposure. Bootstrap analysis based on >1,000 replications was used to derive 95% CIs for the mediated effect [31].

## Results

### Descriptive characteristics

Participants (45.6% men, 41.5% left school aged ≤16 years) were followed for 6.27 ± 0.46 years (mean ± SD). At baseline, 40.4% of participants met the current MVPA guidelines (≥30 min/day), 47.4% were sedentary for ≥8 h/day, 57.3% watched television for ≥2 h/day, 24.0% were normal weight, 47.4% were overweight and 28.6% were obese. As shown in Table 1, mean values for CCMR, CCMR_*no*_ _*adip*_, systolic blood pressure, fasting plasma glucose, fasting serum insulin, sedentary time and television viewing time increased from baseline to 6 years of follow-up. The percentage of wear time spent sedentary increased (58.0 ± 9.5% vs 63.0 ± 9.0%; *p* < 0.001) and percentage of wear time spent in MVPA decreased (3.7 ± 2.3% vs 3.3 ± 2.5%; *p* < 0.05) from baseline to follow-up. Overall, 79% of participants increased their sedentary time and 44% increased their MVPA; 8% changed their smoking behaviour. Compared with women, men had an adverse risk profile for the majority of cardiometabolic risk indicators at baseline and follow-up (Electronic Supplementary Material [ESM] Table 1). Women reported watching more television and engaged in less MVPA but showed lower levels of objectively measured sedentary time compared with men; these sex differences were only statistically significant for sedentary and MVPA time at 6 years of follow-up. Compared with those who were excluded, participants included in the current study were slightly more educated (*p* < 0.05), but were similar in terms of age, BMI, individual cardiometabolic risk factors, sedentary time, MVPA and television viewing time at baseline.

**Table 1:** Descriptive characteristics of participants at baseline and after 6 years of follow-up

Characteristic	Baseline	6 year follow-up	Change
Age (years)	42.5 ± 6.3	48.8 ± 6.3***	6.3 ± 0.5
Body mass index (kg/m^2^)	28.0 ± 4.8	28.0 ± 4.8	0.0 ± 0.2
CCMR	0.0 ± 0.7	0.1 ± 0.8*	0.1 ± 0.5
CCMR_*no*_ _*adip*_	0.0 ± 0.7	0.1 ± 0.8*	0.1 ± 0.5
Waist circumference (cm)	94.2 ± 12.6	94.9 ± 13.5	0.7 ± 7.2
Triacylglycerol (mmol/)	1.3 ± 0.4^a^	1.2 ± 0.4^a^	−0.1 ± 1.1
HDL-cholesterol (mmol/l)	1.4 ± 0.4	1.4 ± 0.4	0.0 ± 0.3
Systolic blood pressure (mmHg)	119.4 ± 12.6	122.7 ± 13.3**	3.3 ± 13.0
Diastolic blood pressure (mmHg)	75.8 ± 9.7	76.3 ± 9.4	0.5 ± 9.2
Fasting plasma glucose (mmol/l)	5.0 ± 0.1^a^	5.3 ± 0.2^a^***	0.4 ± 1.4
Fasting serum insulin (pmol/l)	54.4 ± 0.8^a^	49.5 ± 1.0^a^*	−1.0 ± 41.5
Sedentary time (h/day)	7.9 ± 1.5	9.1 ± 1.7***	1.2 ± 1.7
MVPA (h/day)	0.5 ± 0.3	0.5 ± 0.4	0.0 ± 0.4
Television viewing time (h/day)	2.4 ± 1.1	2.6 ± 1.2***	0.3 ± 0.9
Monitor wear time (h/day)	13.6 ± 1.3	14.5 ± 1.4***	0.9 ± 1.7

Data are means ± SD, unless otherwise indicated

^a^Geometric mean ± SD

*p < 0.05; **p < 0.01; ***p < 0.001 between baseline and follow-up

Television viewing constituted 30.7 ± 14.5% and 29.8 ± 14.2% of objectively measured sedentary time at baseline and follow-up, respectively. Television viewing, sedentary and MVPA time showed relatively weak correlations at both time points (Spearman’s rho: sedentary-MVPA: baseline: −0.18; follow-up: −0.19; sedentary-television: baseline: 0.15; follow-up: 0.05; MVPA-TV: baseline: −0.12; follow-up: −0.26). Correlations between change in exposure variables from baseline to follow-up were also relatively weak (sedentary-MVPA: −0.15; sedentary-TV: −0.05; MVPA-TV: −0.13). All three variables showed moderate tracking, as indicated by correlations between their baseline and follow-up levels (sedentary: 0.49; MVPA: 0.51; television: 0.68) [32].

### Multiple linear regression analysis

Unstandardised and standardised regression coefficients are shown in Table 2 and ESM Table 2, respectively. Greater increase in sedentary time was independently associated with a larger increase in CCMR, CCMR_*no*_ _*adip*_, waist circumference and triacylglycerol after adjusting for relevant confounders in Model A, albeit not quite reaching statistical significance for triacylglycerol (*p* = 0.051). Adjustment for baseline and change in MVPA (Model B) attenuated the association for waist circumference, but not for CCMR, CCMR_*no*_ _*adip*_ and triacylglycerol. Change in waist circumference and change in BMI did not mediate the associations with CCMR_*no*_ _*adip*_ and triacylglycerol (a*b [95% CI]: waist circumference: CCMR_*no*_ _*adip*_: 0.03 [−0.01, 0.07], triacylglycerol: 0.01 [−0.01, 0.04]; BMI: CCMR_*no*_ _*adip*_: 0.01 [−0.01, 0.02], triacylglycerol: 0.03 [−0.02, 0.08]).

**Table 2:** Associations between change in sedentary time, MVPA and television viewing time (all h/day) over 6 years and changes in cardiometabolic risk from baseline to 6 year follow-up

Cardiometabolic outcome	Model	Sedentary time	MVPA time	Television viewing time
CCMR^a,b,c^	A	0.11 (0.04, 0.17)**	−0.33 (−0.57, −0.09)**	0.09 (0.01, 0.19)*
	B	0.08 (0.01, 0.15)*	−0.20 (−0.46, 0.05)	0.08 (−0.01, 0.18)
CCMR_*no*_ _*adip*_^a,b,c^	A	0.10 (0.03, 0.17)**	−0.31 (−0.57, −0.06)*	0.09 (−0.01, 0.19)
	B	0.08 (0.01, 0.16)*	−0.19 (−0.46, 0.09)	0.08 (−0.03, 0.18)
Waist circumference (cm)	A	1.39 (0.44, 2.33)**	−5.17 (−8.60, −1.74)**	1.49 (0.13, 2.86)*
	B	0.93 (−0.08, 1.95)	−3.86 (−7.58, −0.14)*	1.24 (−0.14, 2.63)
Triacylglycerol^a^ (mmol/l)	A	0.13 (−0.01, 0.26)	−0.01 (−0.48, 0.48)	0.11 (−0.08, 0.29)
	B	0.15 (0.01, 0.29)*	0.21 (−0.31, 0.72)	0.10 (−0.09, 0.30)
HDL-cholesterol^a^ (mmol/l)	A	−0.01 (−0.04, 0.02)	0.09 (−0.02, 0.21)	−0.01 (−0.06, 0.03)
	B	−0.01 (−0.04, 0.03)	0.09 (−0.04, 0.21)	−0.01 (−0.06, 0.04)
Systolic blood pressure^b^ (mmHg)	A	0.52 (−1.04, 2.07)	0.81 (−4.96, 6.58)	−0.07 (−2.31, 2.17)
	B	0.73 (−0.97, 2.43)	2.16 (−4.11, 8.43)	−0.14 (−2.48, 2.21)
Diastolic blood pressure^b^ (mmHg)	A	0.70 (−0.37, 1.77)	−1.63 (−5.57, 2.31)	−0.08 (−1.63, 1.48)
	B	0.61 (−0.56, 1.77)	−0.69 (−4.98, 3.61)	−0.20 (−1.81, 1.42)
Fasting plasma glucose^c^ (mmol/l)	A	0.01 (−0.01, 0.01)	−0.02 (−0.06, 0.02)	0.02 (0.01, 0.03)*
	B	0.01 (−0.01, 0.01)	−0.02 (−0.06, 0.02)	0.01 (−0.01, 0.03)
Fasting serum insulin^c^ (pmol/l)	A	4.80 (−0.53, 10.12)	−21.57 (−40.92, −2.23)*	7.68 (0.02, 15.34)*
	B	2.85 (−2.91, 8.62)	−17.25 (−38.34, 3.84)	7.24 (−0.63, 15.10)

Data are unstandardised regression coefficients (95% CI)

Model A: adjusted for baseline age, sex, SES, exposure under study and cardiometabolic risk variable under study; baseline and change in smoking status; change in monitor wear time and in medication for ^a^dyslipidaemia, ^b^hypertension or ^c^dysglycaemia; and follow-up time

Model B: adjusted for all covariates in Model A plus baseline and change in MVPA/sedentary time

*p < 0.05; **p < 0.01

Greater decrease in MVPA time was associated with a larger increase in CCMR, CCMR_*no*_ _*adip*_, waist circumference and fasting serum insulin, independent of relevant confounders in Model A. All of these associations, except for waist circumference, were attenuated after adjustment for baseline and change in sedentary time (Model B).

Although a greater increase in television viewing time was associated with a greater increase in CCMR, waist circumference, fasting plasma glucose and fasting serum insulin in Model A, these associations were all attenuated after adjustment for objectively measured baseline and change in MVPA (Model B).

**Fig. 1:**
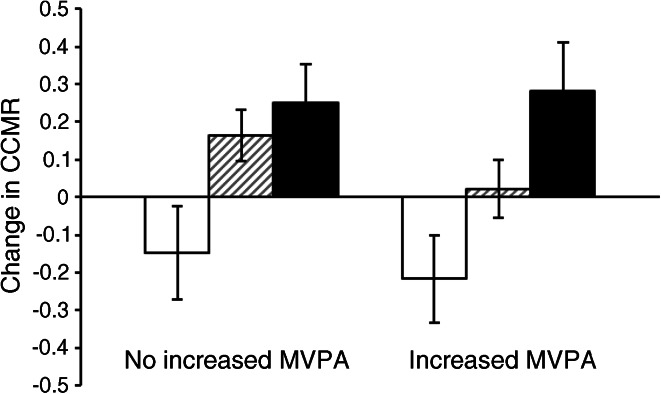
Change in clustered cardiometabolic risk according to change in sedentary time (three groups) and change in MVPA time (two groups). Data are estimated marginal means (SE), adjusted for baseline time spent sedentary, baseline MVPA time, baseline clustered cardiometabolic risk, age, sex and SES; baseline and change in smoking status; change in monitor wear time and in medication for dyslipidaemia, hypertension and dysglycaemia; and follow-up time. White bars represent participants who decreased their sedentary time; grey striped bars represent those who increased their sedentary time by <2 h/day; and black bars represent those who increased their sedentary time by ≥2 h/day. The three left bars represent participants who did not increase their MVPA time, while the three right bars represent participants who increased their MVPA time. Change is follow-up minus baseline

Figure 1 displays the estimated marginal means (SE) for change in CCMR in six groups of participants defined by their change in sedentary time (decreased sedentary time [21%], increased sedentary time by <2 h/day [54%], increased sedentary time by ≥2 h/day [25%]) and their change in MVPA (did not increase MVPA [56%], increased MVPA [44%]). There was no interaction between change in sedentary and MVPA times and change in CCMR. Assuming causality, this suggests that the effect of changing sedentary time on CCMR would be similar among participants who did and did not increase their MVPA during follow-up. In addition, no interaction was found between change in sedentary and MVPA times and change in CCMR_*no*_ _*adip*_ or triacylglycerol.

## Discussion

Increasing sedentary time was independently associated with increasing clustered cardiometabolic risk and triacylglycerol over 6 years in adults with a parental history of type 2 diabetes. Increased MVPA was associated with reduced waist circumference. As one of the first longitudinal studies to examine the associations between sedentary time and cardiometabolic risk in adults at high diabetes risk, we have highlighted the potential benefits of targeting a reduction in sedentary time in future interventions. In context, based on our findings for triacylglycerol levels, decreasing sedentary time by 2 h/day would be associated with an approximately 7% lower risk of cardiovascular events [33]. This is a feasible reduction in sedentary time, as indicated by a recent lifestyle intervention [34].

Our findings extend previous cross-sectional findings in other adult populations of the independent associations between sedentary time and clustered cardiometabolic risk [35] and triacylglycerol levels [6]. The association with change in HDL-cholesterol did not reach statistical significance in our study, although it was in the hypothesised direction, which is consistent with cross-sectional findings from the Health Survey for England [7]. It is, however, in disagreement with the National Health and Nutrition Examination Survey, in which a significant cross-sectional association was found with HDL-cholesterol (albeit most strongly in men and non-Hispanic whites) [6], as well as with the findings from Cooper et al showing a prospective association with HDL-cholesterol in English adults with newly diagnosed type 2 diabetes [14]. Evidence that sedentary time might influence waist circumference and glucose metabolism is also mixed, in both cross-sectional [6, 7, 23, 35–37] and longitudinal studies [14, 17, 38–40]. Although physiological mechanisms need further clarification [19], some of the inconsistent findings to date may, in part, be explained by differences in the populations studied (e.g. health risk status, ethnicity [6] and age) as well as differences in the underlying confounding structures. Our non-significant findings for blood pressure are in agreement with the results from most previous studies [6, 7, 23, 35].

Few significant independent associations were found for change in MVPA in our study. This may indicate that solely focusing on increasing MVPA, a potentially challenging lifestyle target for high-risk individuals [4], may not be the most effective strategy for future interventions [23]. The lack of associations may, however, be explained to some extent by the limited change in MVPA between baseline and follow-up. The relatively small sample size may also partly account for the lack of significant findings, not only for MVPA, but also for the other exposure variables. Nevertheless, even though our findings were not statistically significant, they were generally in the hypothesised directions. In addition, the strengths of the associations are consistent with those from previous studies. Healy et al, for example, showed that sedentary time was more strongly associated with triacylglycerol levels than with HDL-cholesterol levels, and with serum insulin as compared with fasting plasma glucose [6]. Change in central obesity is a potential mediator [20], but we found no evidence that these associations were mediated by changes in general and central adiposity. An alternative biologically plausible mechanism underpinning the detrimental effects of prolonged sitting on triacylglycerol involves reduced lipoprotein lipase activity as a result of fewer skeletal muscle contractions [41].

Individuals at high risk of diabetes enrolled in lifestyle intervention programmes demonstrate limited success in increasing their MVPA levels [4]. Replacing sedentary time with light-intensity activities that are part of daily living might be an important intervention target. The vast majority of the waking day is spent either sedentary or in light-intensity activity [35], and changes in time spent sedentary and in light-intensity activity over 6 years were highly correlated (−0.47). This population thus makes most of its transfers in terms of time use between activities of light and sedentary intensity. The importance of total daily physical activity, rather than MVPA alone, on cardiometabolic risk has been shown before, specifically in high-risk adults [23].

The significant associations between change in television viewing and clustered cardiometabolic risk, waist circumference, fasting plasma glucose and serum insulin were attenuated after adjustment for objectively measured MVPA, indicating they are not independent of MVPA. This may suggest that previous associations between television viewing time and cardiometabolic health, which adjusted for MVPA measured by self-report and were sometimes restricted to leisure-time MVPA only [5, 8, 15, 16, 18, 42], may have been affected by residual confounding for this key variable. Future investigations of the health implications of sedentary behaviour are therefore encouraged to use objective measures of physical activity.

The differential associations for television viewing and sedentary time with cardiometabolic risk found in the current study indicate that findings for television viewing should not be generalised to overall sedentary time. Television viewing in this population represented less than a third of objectively measured sedentary time and correlations between both indicators were weak. In contrast, two previous investigations showed stronger associations between self-reported television viewing time and cardiometabolic risk compared with objectively measured sedentary time, albeit derived from cross-sectional data [7, 36]. A study in adults aged ≥60 years [36], who on average watched more television than younger age groups, showed stronger associations between television viewing and objectively measured sedentary time compared with younger age groups [43]. Future large longitudinal cohort studies covering a wide age range, and also including a whole set of cardiometabolic risk indicators and appropriately accounting for confounding factors, are needed to further explore the differential role of television viewing and sedentary time in determining cardiometabolic health.

To the best of our knowledge, this is the first longitudinal study simultaneously examining the associations of total sedentary time, television viewing time and MVPA time with clustered cardiometabolic risk and a wide set of individual risk factors. All risk factors were used continuously, which optimised statistical power. It is unclear whether participants changed their sedentary time before any changes in cardiometabolic risk, which hampers inferences on the direction of causality. Change in waist circumference has been shown to predict an increase in sedentary time in a healthy, middle-aged white population [40]. However, unlike most previous research, our change models examined within-participant effects, which are less prone to unmeasured time-independent confounders (e.g. genetic factors) because each individual acts as his/her own control. Other major strengths of this study include the objective measurement of two of the three exposures, which substantially reduced measurement error. Participants were followed for longer than 6 years. Finally, the mediation effect of general and central adiposity was also explored using the product of coefficient method [31].

The following limitations should, however, be considered. Participants included in the current study were slightly more educated than those who were excluded, but did not differ in terms of cardiometabolic, sedentary or activity profile, BMI, sex or age. Nevertheless, the relatively small sample of white adults with a high risk for type 2 diabetes included in this study might limit the external validity of the observed associations. Future longitudinal studies in more heterogeneous populations should explore the relative importance of a change in these three behaviours for cardiometabolic health. Television viewing time was self-reported, which confers greater measurement error and is likely to have attenuated any associations, compared with objectively measured exposures. However, assuming participants’ misreporting behaviour would remain constant between baseline and follow-up, a change in television viewing should be captured with lower measurement error compared with television viewing time measured at just one time point.

Participants wore the accelerometer for 4 consecutive days, not necessarily including a weekend day. Sedentary and MVPA time might therefore not be completely reflective of participants’ habitual long-term behaviour. Despite controlling for several potential confounding variables, some findings could still be partially explained by other factors that were not measured, such as alcohol intake, employment status and type of work, or only partially measured (e.g. SES by means of age at finishing full-time education). Given the lack of dietary information, the mediating role of snacking and overall dietary quantity and quality, which are especially relevant for television viewing, could not be explored. Finally, to examine our research question, we conducted a number of significance tests, which might have increased the risk of type 1 errors. Future longitudinal research will help to confirm our findings.

In conclusion, in adults at high risk for type 2 diabetes, increasing sedentary time was independently associated with increasing clustered cardiometabolic risk and triacylglycerol levels. Replacing sedentary time by light-intensity activity, and thus increasing overall daily physical activity levels, could be an important behavioural target in these high-risk individuals. Further longitudinal observational studies are now needed to extend these findings to other populations that are heterogeneous in terms of age, ethnicity and health status, as well as intervention studies, both physiological and free-living, examining acute and longer-term effects to further investigate the direction and mechanism of causality.

## Electronic supplementary material

Below is the link to the electronic supplementary material.

ESM Table 1 (PDF 11 kb)

ESM Table 2 (PDF 11 kb)

## Abbreviations

CCMR: Clustered cardiometabolic risk score
CCMR_*no*__*adip*_: Clustered cardiometabolic risk score without adiposity component
MET: Metabolic equivalent
MVPA: Moderate-to-vigorous physical activity
SES: Socioeconomic status
