# Supplementary material for: Increasing objectively measured sedentary time increases clustered cardiometabolic risk: a 6 year analysis of the ProActive study
Source: Diabetologia. 2013 Nov 6;57(2):305–12. doi: 10.1007/s00125-013-3102-y (PMC3889989; doi:10.1007/s00125-013-3102-y)
Supplement: Supplementary file 1 — (PDF 11 kb) [file 125_2013_3102_MOESM1_ESM.pdf]

Supplementary Table S1

Table S1. Descriptive characteristics of men and women at baseline and 6-year follow-up, *ProActive*

	Men (n=78)		Women (n=93)	
Characteristic	Baseline	6-year follow-up	Baseline	6-year follow-up
Age, years	42.0 (6.2)	48.3 (6.2) [d]	43.0 (6.3)	49.2 (6.3) [d]
Body mass index, kg/m^2	27.9 (4.0)	27.9 (4.0)	28.1 (5.4)	28.1 (5.4) [b]
CCMR	0.0 (0.7)	0.0 (0.7)	0.0 (0.7)	0.1 (0.9)
$CCMR_{no\ adip}$	0.0 (0.7)	0.1 (0.7)	0.0 (0.7)	0.1 (0.9)
Waist circumference, cm	99.8 (10.1)	99.1 (11.2)	89.5 (12.5) [g]	91.3 (14.3) [b g]
Triacylglycerol [a], mmol/ l	1.5 (0.5)	1.5 (0.4)	1.1 (0.3) [g]	1.0 (0.4) [g]
HDL-cholesterol, mmol/ l	1.2 (0.3)	1.2 (0.2)	1.6 (0.4) [g]	1.6 (0.4) [g]
Systolic blood pressure, mmHg	123.4 (10.7)	124.8 (11.2)	116.0 (13.2) [g]	120.9 (14.6) [c]
Diastolic blood pressure, mmHg	78.3 (8.7)	76.9 (9.4)	73.7 (10.1) [f]	75.9 (9.3) [b]
Fasting plasma glucose [a], mmol/ l	5.2 (0.2)	5.6 (0.3) [c]	4.8 (0.1) [g]	5.0 (0.1) [d f]
Fasting serum insulin [a], pmol/ l	60.2 (0.7)	57.3 (0.9)	49.9 (0.8) [e]	43.7 (1.1) [b e]
Sedentary time, hours/day	8.0 (1.6)	9.6 (1.8) [d]	7.8 (1.4)	8.8 (1.6) [d f]
MVPA, hours/day	0.5 (0.3)	0.6 (0.4)	0.5 (0.3)	0.4 (0.3) [f]
TV viewing time, hours/day	2.3 (1.0)	2.5 (1.0) [b]	2.5 (1.2)	2.8 (1.3) [c]
Monitor wear time, hours/day	13.8 (1.3)	14.9 (1.4) [d]	13.5 (1.3)	14.1 (1.3) [d g]

Data are means (SD). [a] Geometric mean (SD).
Abbreviations: CCMR: clustered cardio-metabolic risk score including waist circumference; $CCMR_{no\ adip}$: clustered cardio-metabolic risk score excluding waist circumference; MVPA: moderate-to-vigorous physical activity.
[b] $p<0.05$; [c] $p<0.01$; [d] $p<0.001$ between baseline and follow-up; [e] $p<0.05$; [f] $p<0.01$; [g] $p<0.001$ between men and women.
