# Supplementary material for: Increasing objectively measured sedentary time increases clustered cardiometabolic risk: a 6 year analysis of the ProActive study
Source: Diabetologia. 2013 Nov 6;57(2):305–12. doi: 10.1007/s00125-013-3102-y (PMC3889989; doi:10.1007/s00125-013-3102-y)
Supplement: Supplementary file 2 — (PDF 11 kb) [file 125_2013_3102_MOESM2_ESM.pdf]

Supplementary Table S2

Table S2. Associations between change in sedentary time (hours/day), moderate-to-vigorous physical activity (MVPA; hours/day) and TV viewing time (hours/day) over 6 years with changes in cardio-metabolic risk from baseline to six year follow-up, standardised regression coefficients

Cardio-metabolic outcome	Model	Sedentary time	MVPA time	TV viewing time
CCMR [a b c]	A	0.37 (0.15, 0.59) [e]	-0.24 (-0.42, -0.07) [e]	0.17 (0.01, 0.34) [d]
	B	0.29 (0.05, 0.53) [d]	-0.15 (-0.34, 0.04)	0.15 (-0.03, 0.32)
$CCMR_{no\ adip}$ [a b c]	A	0.33 (0.11, 0.55) [e]	-0.21 (-0.39, -0.04)	0.15 (-0.02, 0.32)
	B	0.26 (0.02, 0.50) [d]	-0.13 (-0.32, 0.06)	0.13 (-0.04, 0.30)
Waist circumference	A	0.33 (0.10, 0.55) [e]	-0.26 (-0.43, -0.09) [e]	0.18 (0.02, 0.35) [d]
	B	0.22 (-0.02, 0.46)	-0.19 (-0.38, -0.01) [d]	0.15 (-0.02, 0.32)
Triacylglycerol [a]	A	0.20 (-0.01, 0.41)	-0.01 (-0.16, 0.16)	0.09 (-0.07, 0.24)
	B	0.23 (0.01, 0.45) [d]	0.07 (-0.11, 0.25)	0.09 (-0.08, 0.25)
HDL-cholesterol [a]	A	-0.07 (-0.28, 0.13)	0.13 (-0.03, 0.28)	-0.05 (-0.20, 0.11)
	B	-0.01 (-0.23, 0.21)	0.12 (-0.05, 0.30)	-0.03 (-0.19, 0.13)
Systolic blood pressure [b]	A	0.07 (-0.14, 0.27)	0.02 (-0.14, 0.18)	-0.01 (-0.16, 0.15)
	B	0.10 (-0.13, 0.32)	0.06 (-0.12, 0.24)	-0.01 (-0.17, 0.15)
Diastolic blood pressure [b]	A	0.13 (-0.07, 0.33)	-0.06 (-0.22, 0.09)	-0.01 (-0.16, 0.14)
	B	0.11 (-0.10, 0.33)	-0.03 (-0.20, 0.14)	-0.02 (-0.18, 0.14)
Fasting plasma glucose [c]	A	0.05 (-0.16, 0.26)	-0.09 (-0.25, 0.07)	0.16 (0.01, 0.31) [d]
	B	0.01 (-0.22, 0.23)	-0.08 (-0.26, 0.09)	0.14 (-0.02, 0.30)
Fasting serum insulin [c]	A	0.20 (-0.02, 0.41)	-0.19 (-0.36, -0.02) [d]	0.16 (0.01, 0.33) [d]
	B	0.12 (-0.12, 0.35)	-0.15 (-0.33, 0.03)	0.15 (-0.01, 0.32)

Data are standardised regression coefficients (95% CI) following standardisation of exposure and outcome variables. Statistically significant (P <0.05) estimates are indicated in bold.

Model A: adjusted for baseline age, gender, socio-economic status, exposure under study and cardio-metabolic risk variable under study; baseline and change in smoking status; change in monitor wear time and in medication for dyslipidaemia [a]/hypertension [b]/dysglycaemia[c]; and follow-up time; Model B: adjusted for all covariates in Model A plus baseline and change in MVPA/sedentary time.

Abbrevations: CCMR: clustered cardio-metabolic risk score including waist circumference; $CCMR_{no\ adip}$: clustered cardio-metabolic risk score excluding waist circumference.

[d] p<0.05; [e] p<0.01
